# The Book World of Medicine and Science

**Published:** 1897-07-03

**Authors:** 


### The Book World of Medicine and Science.

The White Slaves of England. By R. H. Sherard. Illustrated by Harold Piffard. (London: James Bowman. 1897.)

This is an interesting book in two senses. On the one hand if it is to be believed it shows the extremely hard conditions under which many of the workers of England perform their daily tasks; on the other it shows the hopelessness of getting at the truth of the labour problem so long as those who write upon the subject do so in complete defiance of, and apparently in complete ignorance of, the well-recognised facts of political economy. Even if we grant that the writer has not been imposed upon, and that what he tells us is a true description of the facts of the case, we cannot but see that the conditions of labour which he describes are the result not, as he suggests, of the tyranny of the masters, but of the competition of man with man to snatch the work from one another. Still those who choose to look for it can find the truth even in this volume. The reason of low wages is the competition that exists even for such wages as are offered. The following statement by a Bradford woolcomber is given as showing the harsh treatment of the operatives by the masters, but to our mind it shows exactly where the shoe pinches, and that is in the superabundance of unskilled labour. The workman said, "If a man is five minutes late at our shop he's sent right off. Says the gaffer, 'If you don't want to earn your living go to blazes. There's plenty here as does.'" There is the whole matter in a nutshell, and a writer who does not see that can hardly be accepted as an authority on the subject. We have every sympathy with those whose lot it is to labour under the conditions described in this book. The fate of the chainmakers, for example, seems a hard one. Nevertheless, we come across sentences here and there which seem to hint that a good deal lies in the hands of the men themselves. The pay may not be good, but when we hear that "beer plays a great part in the lives of the men," and that they often consume three shillingsworth of beer a day, we cannot but feel that there is another side to the question, a side which Mr. Sherard has not touched upon. When we recognise with what docility workmen follow the orders of those who control their trades unions, and how readily they come out on strike in reference to miserable questions of odd farthings in wages or of the employment of workmen to do work which is not sanctioned by their rules, and when one sees how immensely powerful the unions are when they choose to put forth their strength, we cannot but feel that they have in their own hands the power to remove many of the evils mentioned in this book if they cared to do so. Unfortunately, the unions tend to measure everything by wages. The men who pack chloride of lime in the alkali works undoubtedly do work which is both injurious to health and dangerous to life. It seems to be absolutely unskilled labour, yet by it they can earn as much as fifty shillings a week. Now it is perfectly well known that by means of a sort of diving dress, into the mask of which fresh air can be pumped from outside, most of the danger can be removed. But would the men get fifty shillings a week for the work if such apparatus were introduced in o English works and the danger were thus removed? That is where the rub comes. The economics of the labour problem touch upon many questions which are not to be solved by sympathy alone. Some amount of knowledge is necessary, and some amount of willingness to see the other side. Both of these qualities are strikingly absent from the volume before us. Nevertheless, the author has shown himself to be capable of telling a striking story and making a good case, which is, perhaps, the main point.

